# Surveying bovine digital dermatitis and non-healing bovine foot lesions for the presence of *Fusobacterium necrophorum, Porphyromonas endodontalis* and *Treponema pallidum*

**DOI:** 10.1136/vr.105628

**Published:** 2020-02-17

**Authors:** Gareth James Staton, Leigh Emma Sullivan, Roger W Blowey, Stuart D Carter, Nicholas James Evans

**Affiliations:** 1 Institute of Infection & Global Health, University of Liverpool, Neston, Cheshire, UK; 2 Wood Veterinary Group, Gloucester, UK

**Keywords:** microbiology, lameness, cattle

## Abstract

**Background:**

Non-healing bovine foot lesions, including non-healing white line disease, non-healing sole ulcer and toe necrosis, are an increasingly important cause of chronic lameness that are poorly responsive to treatment. Recent studies have demonstrated a high-level association between these non-healing lesions and the *Treponema* phylogroups implicated in bovine digital dermatitis (BDD). However, a polymicrobial aetiology involving other gram-stain-negative anaerobes is suspected.

**Methods:**

A PCR-based bacteriological survey of uncomplicated BDD lesions (n=10) and non-healing bovine foot lesions (n=10) targeting *Fusobacterium necrophorum*, *Porphyromonas endodontalis, Dichelobacter nodosus* and *Treponema pallidum/T. paraluiscuniculi* was performed.

**Results:**

*P. endodontalis* DNA was detected in 80.0% of the non-healing lesion biopsies (p=<0.001) but was entirely absent from uncomplicated BDD lesion biopsies. When compared to the BDD lesions, *F. necrophorum* was detected at a higher frequency in the non-healing lesions (33.3% vs 70.0%, respectively), whereas *D. nodosus* was detected at a lower frequency (55.5% vs 20.0%, respectively). Conversely, *T. pallidum/T. paraluiscuniculi* DNA was not detected in either lesion type.

**Conclusion:**

The data from this pilot study suggest that *P. endodontalis* and *F. necrophorum* should be further investigated as potential aetiological agents of non-healing bovine foot lesions. A failure to detect syphilis treponemes in either lesion type is reassuring given the potential public health implications such an infection would present.

## Introduction

Bovine digital dermatitis (BDD), an inflammatory disease of the interdigital skin with an infectious aetiology, is one of the most frequently encountered causes of lameness.^1^ Conversely, important primary causes of non-infectious bovine lameness include white line disease (WLD) and sole ulcer (SU), both of which affect the horn.^2^ Over the last 15 years, anecdotal reports of new bovine foot disorders have increased.^3^ In particular, disorders that grossly resemble WLD and SU but exhibit a more aggressive clinical phenotype appear to be increasing. The prefix ‘non-healing’ has been adopted to distinguish these disorders from their classical presentations; toe necrosis (TN) has also been included in this category. By definition, these lesions are refractory to conventional therapies and require prolonged treatment or amputation of the diseased claw.^4^ Importantly, non-healing (nh)WLD, nhSU and TN appear to be epidemiologically associated with BDD and exhibit similarities in gross pathology, including a moist, granular topical appearance and pungent malodour.^5^ Molecular studies strongly support the involvement of specific treponeme phylogroups (*Treponema medium* phylogroup, *Treponema phagedenis* phylogroup and *Treponema pedis*) in the aetiology of BDD and the non-healing lesions.^3 6–8^ However, both BDD and its ovine variant, contagious ovine digital dermatitis (CODD), involve polymicrobial infections in which the BDD-associated treponemes may be necessary but not sufficient for disease.^9 10^ Similarly, the aetiologies of non-healing bovine foot lesions are also likely to involve multiple bacterial species, the characterisation of which may explain the enhanced lesion severity.

To investigate this possibility further, non-healing bovine foot lesion biopsies were surveyed for the two other key bacterial species known to be associated with ruminant foot disease, namely *Dichelobacter nodosus* and *Fusobacterium necrophorum*, the aetiological agents of ovine footrot,^11 12^ which are also pathologically associated with CODD^13^ and BDD.^14^ In addition, the *Porphyromonas* genera is highly associated with BDD lesions^15–17^ and because *Porphyromonas endodontalis* has previously been detected in 80% of surveyed CODD lesions,^18^ the presence of this species in non-healing lesions was investigated. Finally, because the changes in bone density reported in TN^19^ histologically resemble syphilitic osteoporosis^20^ and BDD lesions histologically resemble the exudative papillomatous lesions of Yaws,^21^ we considered it prudent to screen both the BDD and non-healing lesion biopsies for their respective aetiological agents, *Treponema pallidum subs. pallidum* and *T. pallidum* subs. *pertenue* DNA. This PCR was also designed to detect *Treponema paraluiscuniculi*, the aetiological agent of venereal syphilis in rabbits, which has previously been identified in BDD lesion microbiome datasets.^22^

## Materials and methods

The non-healing lesion samples used here were collected between February and July 2009 from nine Holstein Friesian cows living on nine dairy farms in the UK (seven in Gloucestershire and two in Cambridgeshire) and comprised of nhWLD (n=3), nhSU (n=3) and TN (n=4) lesion punch biopsies. Samples were processed as described previously.^7^ Similarly, the BDD lesion samples were collected between December 2003 and November 2006, from 10 Holstein Friesian cows living on 8 dairy farms in the UK (two in Merseyside, one in Shropshire, three in Gloucestershire and two in Cheshire), as previously described.^8^ All biopsied BDD lesions were classified as ‘M2’ (ulcerative) grade lesions^23^ by the attending clinician. Sampling was performed under Home Office project license PPL 40/2574.

All lesion biopsy samples were subjected to the following PCR assays, performed as described previously: *F. necrophorum*,^24^ *D. nodosus* 13 and *P. endodontalis*.^25^ The primers used in this study are shown in table 1. All PCRs were performed on a Mastercycler Gradient thermocycler (Eppendorf, Germany). A PCR targeting the *T. pallidum* 16S rRNA gene was developed as part of this study. The *T. pallidum* PCR thermal profile was as follows: an initial denaturation at 95°C for 7 min followed by 35 cycles of 95°C for 1 min, 71°C for 2 min and 72°C for 3 min and a final elongation step at 72°C for 10 min. DNA extracted from *T. pallidum* subsp. *pallidum* (Nichol strain, NCID, Atlanta, USA) was used as a positive control. To validate this assay, purified water and genomic DNA from the three BDD-associated *Treponema* phylogroups were used as negative controls. Each 25 µL PCR reaction included 1 µL of DNA template and was performed using *Taq* polymerase (Qiagen, Crawley, UK) in accordance with the manufacturer’s instructions. All PCRs were performed in duplicate with relevant genomic DNA controls. Amplicons were visualised by gel electrophoresis and ethidium bromide staining. Statistical analysis was performed using the Fisher’s exact test in Minitab V.18 (Minitab Inc., PA, USA).

**Table 1:** Primers used in the study

Gene specificity	Species	Primer sequence (5’−3’)	Predicted band size (bp)	Reference
IktA	*F. necrophorum*	F: ACAATCGGAGTAGTAGGTTC R: ATTTGGTAACTGCCACTGC	402	24
16S rRNA	*D. nodosus*	F: TGAAGAATGAAAGCGGGGGC R: CTAATCCTGTTTGCTACCCACG	583	13
16S rRNA	*P. endodontalis*	F: GCTGCAGCTCAACTGTAGTC R: CCGCTTCATGTCACCATGTC	672	25
16S rRNA	*T. pallidum*	F: CGCGTGGGTAATCTGCCTTT R: TTTCTACGGCGCTCCTCTTGA	903	This study

F, forward primer; R, reverse primer.

## Results

The dataset (table 2) shows the presence (+) or absence (−) of specific PCR products as determined by relevant bacterial diagnostic assays.

**Table 2:** PCR detection of fastidious gram-stain-negative anaerobes in BDD and bovine non-healing foot lesions

Sample no.	Biopsy date	Lesion type	*F. necrophorum*	*D. nodosus*	*BDD Treponema* phylogroup			*Treponema* genus	*P*. *endodontalis*	*T*. *pallidum*
					1	2	3			
1	24-February-09	nhWLD*	−	−	+	+	+	+	−	−
2	03-March-09	nhWLD*	−	−	+	+	+	+	+	−
3	09-March-09	nhWLD*	+	−	+	+	+	+	+	−
4	09-March-09	TN*	+	−	+	+	+	+	+	−
5	03-April-09	TN*	+	−	+	+	+	+	+	−
6	17-March-09	TN*	+	−	+	+	+	+	+	−
7	19-March-09	TN*	−	+	+	+	+	+	+	−
8	16-March-09	nhSU*	+	−	+	−	+	+	+	−
9	14-July-09	nhSU*	+	−	+	+	+	+	+	−
10	14-July-09	nhSU*	+	+	−	−	−	+	−	−
11	09-July-04	BDD†	−	−	+	+	+	+	−	−
12	26-January-04	BDD†	+	−	+	+	+	+	−	−
13	23-April-04	BDD†	nd	nd	+	+	-	+	−	−
14	16-May-04	BDD†	−	+	+	+	+	+	−	−
15	26-January-04	BDD†	+	+	+	+	+	+	−	−
16	02-September-05	BDD†	−	−	+	+	−	+	−	−
17	13-February-04	BDD†	−	+	+	+	+	+	−	−
18	26-April-04	BDD†	+	+	+	+	+	+	−	−
19	01-December-03	BDD†	−	+	+	+	+	+	−	−
20	26-April-04	BDD†	−	−	+	+	+	+	−	−

*Non-healing lesion *Treponema* phylogroup PCR results and *Treponema* genus PCR results previously reported.^3^

†BDD lesion *Treponema* phylogroup PCR results and *Treponema* genus PCR results reported previously.^8^

BDD, bovine digital dermatitis; nhWLD, non-healing white line disease; TN, toe necrosis; nhSU, non-healing sole ulcer; n.d., not determined.

These bacteriological profiles, while confirming the ubiquity of BDD treponemes in both digital dermatitis lesions and non-healing lesions, also reveal certain distinctions. Most strikingly, *P. endodontalis* DNA was found to be highly associated (80.0%, p ≤ 0.001) with the non-healing lesions but entirely absent from typical, uncomplicated BDD lesions. *F. necrophorum* DNA was also detected at a higher frequency in non-healing lesions relative to BDD lesions (70.0% vs 33.3%, respectively), whereas *D. nodosus* was detected at a lower frequency (20.0% vs 55.5%, respectively), although these differences were not statistically significant. Neither *T. pallidum* nor *T. paraluiscuniculi* DNA was detected in either lesion type.

## Discussion

The aetiologies of non-healing bovine foot lesions are poorly understood. A high-level association with the BDD-associated treponemes supports their involvement in the pathogenesis of these lesions, but the roles played by other pathogenic bacteria remains unknown. Based on the data presented here, it is hypothesised that in addition to the BDD treponemes, infection with *F. necrophorum* and *P. endodontalis* may also contribute to pathogenesis of TN, nhWLD and nhSU. *F. necrophorum* is considered to be an opportunistic pathogen and has been implicated in several animal diseases, including interdigital phlegmon,^26^ ovine footrot,^27^ hepatic abscesses^28^ and calf diphtheria.^29^

In humans, *P. endodontalis* is strongly associated with chronic oral infections where it participates in tissue destructive processes.^30 31^ In particular, *P. endodontalis*-derived virulence factors, including lipopolysaccharide, are potent stimulators of inflammatory cytokine release, and are thought to have a role in the initiation and development of periapical periodontitis, odontogenic abscesses and alveolar bone abnormalities.^32 33^ The frequent (80.0%) detection of *P. endodontalis* in the non-healing bovine foot lesions (and its complete absence from BDD lesions) suggests a potential pathogenic role here, too. To the best of our knowledge, *P. endodontalis* colonisation distal to the oral cavity has hitherto only been observed in CODD,^18^ where it plays an undefined role. The data provided here suggest that *P. endodontalis* colonisation may be a prominent feature of aggressive foot lesions in ruminants. We hypothesise that synergy between *P. endodontalis, F. necrophorum* and the BDD-associated treponemes may lead to enhanced lesion pathology. Conversely, no association between the two *T. pallidum* subspecies*, T. paraluiscuniculi* and either lesion type was identified. This is in contrast to the findings of a recent microbiome study that reported the presence of *T. paraluiscuniculi* in the BDD lesion biopsies of North American cattle.^22^ However, since this organism has not been detected in BDD lesion biopsies by others using similar methodologies,^15 34 35^ a pathogenic role for *T. paraluiscuniculi* in BDD is considered improbable.

In summary, *P. endodontalis* and *F. necrophorum* were both detected at a greater frequency in the non-healing bovine foot lesions relative to uncomplicated BDD lesions. Further studies are required to elucidate the precise relationship between these fastidious gram-stain-negative anaerobes and other relevant species of bacteria in the aetiopathogenesis of these atypical lesions. A failure to detect syphilis treponemes in either lesion type is reassuring given the potential public health implications such an infection would present.
